# Changes of PM_2.5_ and O_3_ and their impact on human health in the Guangdong-Hong Kong-Macao Greater Bay Area

**DOI:** 10.1038/s41598-024-62019-w

**Published:** 2024-05-16

**Authors:** Hui Zhao, Zeyuan Chen, Chen Li

**Affiliations:** 1 School of Resources and Environmental Engineering, Jiangsu University of Technology, Changzhou, 213001 China; 2 Department of Environmental Science and Engineering, Fudan University, Shanghai, 200438 China; 3 School of Electronic and Information Engineering, Wuxi University, Wuxi, 214105 China; 4 No.2 High School of East China Normal University, Shanghai, 201203 China

**Keywords:** Ground-level O_3_, PM_2.5_, Health effects, Risk assessment, Environmental impact, Climate-change impacts

## Abstract

In recent years, the combined pollution of PM_2.5_ and O_3_ in China, particularly in economically developed regions such as the Guangdong-Hong Kong-Macao Greater Bay Area (GBA), has garnered significant attention due to its potential implications. This study systematically investigated the changes of PM_2.5_ and O_3_ and their associated human health effects in the GBA, utilizing observational data spanning from 2015 to 2019. The findings revealed a spatial trend indicating a gradual decrease in PM_2.5_ levels from the northwest to the southeast, while the spatial distribution of MDA8 O_3_ demonstrated an opposing pattern to that of PM_2.5_. The monthly fluctuations of PM_2.5_ and MDA8 O_3_ exhibited V-shaped and M-shaped patterns, respectively. Higher MDA8 O_3_ concentrations were observed in autumn, followed by summer and spring. Over the five-year period, PM_2.5_ concentrations exhibited a general decline, with an annual reduction rate of 1.7 μg m^−3^/year, while MDA8 O_3_ concentrations displayed an annual increase of 3.2 μg m^−3^. Among the GBA regions, Macao, Foshan, Guangzhou, and Jiangmen demonstrated notable decreases in PM_2.5_, whereas Jiangmen, Zhongshan, and Guangzhou experienced substantial increases in MDA8 O_3_ levels. Long-term exposure to PM_2.5_ in 2019 was associated with 21,113 (95% CI 4968–31,048) all-cause deaths (AD), 1333 (95% CI 762–1714) cardiovascular deaths (CD), and 1424 (95% CI 0–2848) respiratory deaths (RD), respectively, reflecting declines of 27.6%, 28.0%, and 28.4%, respectively, compared to 2015. Conversely, in 2019, estimated AD, CD, and RD attributable to O_3_ were 16,286 (95% CI 8143–32,572), 7321 (95% CI 2440–14,155), and 6314 (95% CI 0–13,576), respectively, representing increases of 45.9%, 46.2%, and 44.2% over 2015, respectively. Taken together, these findings underscored a shifting focus in air pollution control in the GBA, emphasizing the imperative for coordinated control strategies targeting both PM_2.5_ and O_3_.

## Introduction

Over the past few decades, the state and characteristics of atmospheric pollution in China have undergone a gradual transformation from singular sources of pollution, such as coal smoke and petrochemical emissions, towards a more intricate form of atmospheric pollution^1^. Notably, traditional pollutants such as sulfur dioxide (SO_2_) and total suspended particulate (TSP) have been effectively controlled. However, the rapid increase in the number of motor vehicles has led to a continuous rise in nitrogen oxides (NO_*x*_) emissions^2^, resulting in a progressively severe regional air pollution characterized by high concentrations of fine particulate matter (PM_2.5_) and ground-level ozone (O_3_)^3^. This trend is particularly pronounced in economically developed regions such as the Beijing-Tianjin-Hebei region (BTH), the Yangtze River Delta (YRD), and the Pearl River Delta (PRD)^4,5^.

Among the primary air pollutants, PM_2.5_ and O_3_ are recognized as pivotal contributors to atmospheric compound pollution^6^. PM_2.5_, defined as particulate matter with an aerodynamic diameter of 2.5 µm or less, originates from diverse sources, encompassing both natural and anthropogenic sources^7^. As a secondary pollutant, O_3_ is generated through the photochemical reaction of precursor pollutants such as NOx and volatile organic compounds (VOCs) under sunlight exposure. These precursor emissions predominantly stem from industrial processes, vehicular exhaust, and various anthropogenic activities^8^. As of 2018, the annual mean PM_2.5_ and maximum daily 8 h average concentrations of O_3_ (MDA8 O_3_) in China remained relatively high, with concentrations recorded at 38.4 μg m^−3^ and 95.8 μg m^−3^ respectively^9^. Against the background of global climate change, atmospheric pollution attributed to PM_2.5_ and O_3_ have emerged as a significant environmental and public health concern, given its profound impact on air quality, human health, and the global environment^10–12^.

PM_2.5_ and O_3_ pose significant risks to human health, as they can irritate the respiratory tract, leading to symptoms such as coughing, wheezing, and shortness of breath^13^. These pollutants also have the potential to exacerbate preexisting respiratory conditions such as asthma and chronic obstructive pulmonary disease^13^. Moreover, they can penetrate deep into the lungs, causing inflammation and resulting in lung damage. Prolonged exposure to PM_2.5_ and O_3_ can lead to decreased lung function over time. Furthermore, epidemiological studies have highlighted the association between PM_2.5_ and O_3_ exposure and cardiovascular issues. These pollutants can enter the bloodstream, contributing to the development of heart diseases, including heart attacks, strokes, and hypertension^14^. Utilizing observed PM_2.5_ data, Maji et al.^15^ reported that in China, PM_2.5_-related hospital admissions due to respiratory and cardiovascular diseases in 2016 were 610,000 (95% CI 370,000–860,000) and 360,000 (95% CI 200,000–520,000), respectively. Additionally, the total morbidity estimates for asthma attack, chronic bronchitis, and emergency hospital admissions were 1,000,000 (95% CI 700,000–1,280,000), 990,000 (95% CI 500,000–1,440,000), and 120,000 (95% CI 60,000–180,000), respectively^15^. In a separate study, Zhao et al^16^ employed meta-analysis method techniques to estimate O_3_-related health effects across China in 2018. Their findings revealed that the total number of all-cause, cardiovascular, and respiratory deaths attributable to O_3_ were 178,529 (95% CI 90,584–346,912), 118,842 (95% CI 40,787–192,507), and 38,178 (95% CI 0–80,159), respectively.

In recent decades, the Chinese government has demonstrated a steadfast commitment to monitoring and mitigating air pollution, instituting a series of policies and measures aimed at enhancing air quality. Previous studies have indicated that the successful implementation of the “Air Pollution Prevention and Control Action Plan” since 2013 has resulted in a decline in PM_2.5_ levels across China, with a reduction rate of 3.4 μg m^−3^ per year, particularly notable in regions such as BTH, central China, and northeast China had larger declines^17^. However, there has been a notable upward trend in the national average O_3_ concentration, showing an annual increase of 3.4 μg m^−3^ per year, with more pronounced increases observed certain regions, including PRD^17^^.^ It is noteworthy that while PM_2.5_ levels have decreased nationally, no significant change has been observed in the PRD, suggesting that PM_2.5_ pollution in this area may still be at high levels^9,17^. The Guangdong-Hong Kong-Macao Greater Bay Area (GBA), encompassing nine cities in the PRD, Hong Kong, and Macao, stands as one of China's most economically robust regions. Despite its economic strength, the GBA lags behind other global Greater Bay Areas in terms of air quality^18^. Moreover, there has been increasing attention on atmospheric compound pollution characterized by PM_2.5_ and O_3_ in this region. To date, no comprehensive studies have been conducted to assess their impact on human health^19^.

Given the above concerns, the aim of this study is to examine the spatial distribution and temporal trends of PM_2.5_ and O_3_ in the GBA from 2015 to 2019, and to quantify their impact on human health.

## Data and methods

### Data source

**Figure 1:**
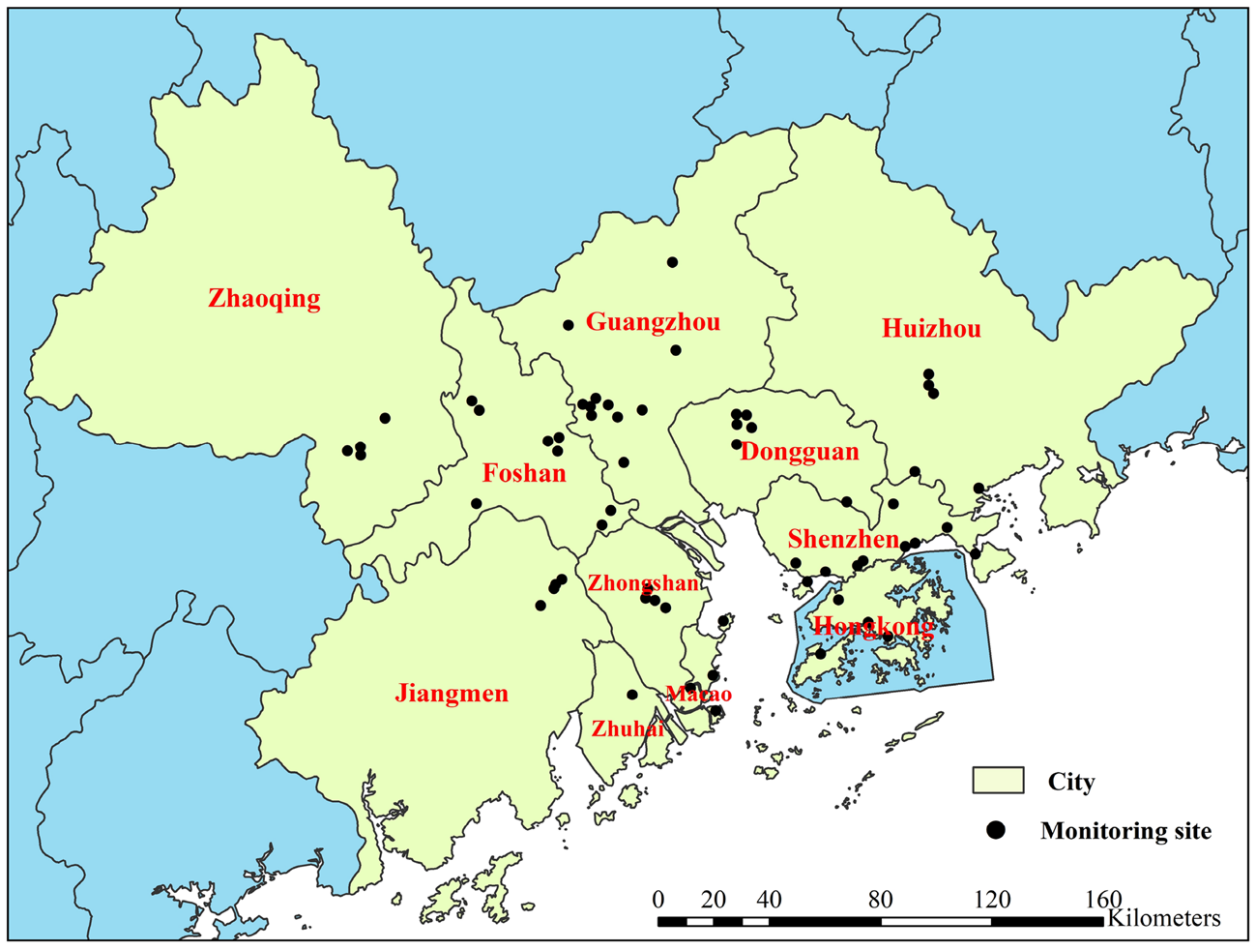
Distribution of air quality monitoring sites in the GBA (The map was generated by ArcGIS 10.7 https://www.esri.com/en-us/arcgis/products/arcgis-desktop/resources).

This study utilized monthly PM_2.5_ and MDA8 O_3_ data from nine cities in the PRD, Hong Kong, and Macao spanning from 2015 to 2019. These data were obtained from two sources: https://quotsoft.net/air/ and the monitoring results reports of the Guangdong-Hong Kong-Macao Pearl River Delta Regional Air Quality Monitoring Network (http://gdee.gd.gov.cn/kqjc/index.html). The combined network comprises 61 air quality automatic monitoring stations, distributed throughout the GBA, including 11 in Guangzhou, 11 in Shenzhen, 8 in Foshan, 4 in Zhuhai and 4 in Jiangmen 4 in Zhaoqing, 5 in Huizhou, 4 in Zhongshan, 5 in Dongguan, 4 in Hong Kong, and 1 in Macao, as illustrated in Fig. 1. The annual mean concentrations of PM_2.5_ and MDA8 O_3_ per station were calculated by averaging the monthly mean values for all months of the year. Subsequently, the annual averaged concentrations for each city were determined based on all stations in this city.

Moreover, population data for the nine cities in the PRD, Hong Kong, and Macao for each year were obtained from the statistical yearbooks of the Guangdong Provincial Bureau of Statistics (http://stats.gd.gov.cn/gdtjnj/), the Hong Kong Census and Statistics Department (https://www.censtatd.gov.hk/sc/), and the Macao Census and Statistics Department (https://www.dsec.gov.mo/zh-MO/).

### Health impact assessment

Many epidemiological studies on air pollution rely on Health Impact Assessment (HIA), a widely employed method for quantify the potential effects of various air pollutants, including PM_2.5_, PM_10_, SO_2_, NO_2_, CO, and O_3_, on human health^13^^.^ HIA involves determining the health risk for an individual when the concentration of a particular air pollutant exceeds a certain threshold, typically calculated based on the exposure–response coefficient (β). It's worth noting that the β value in assessing the health risks associated with long-term exposure to air pollution is typically calculated through epidemiological studies. These studies often analyze extensive population data, including individuals exposed to varying levels of air pollutants and their health outcomes, such as the number of people with cardiovascular or respiratory diseases. By statistically analyzing these data, the association between air pollution exposure and health issues can be determined. The β value is a crucial parameter derived from this association analysis, representing the magnitude of the impact on health risks per unit increase in air pollutant concentration. For PM_2.5_, the β value indicates the relative increase in health risks for each unit increase in PM_2.5_ concentration. For instance, if a study finds that for every 10 μg m^−3^ increase in PM_2.5_ concentration, the incidence of cardiovascular diseases increases by 20%, the corresponding β value would be 0.2. Thus, establishing the exposure–response relationship between air pollutants and mortality is crucial for conducting HIA. Through extensive literature review and and meta-analysis, previous studies have identified associations between PM_2.5_ and premature deaths. Specifically, an annual mean increase of 1 μg m^−3^ in PM_2.5_ concentration was found to correspond to 0.34%, 0.07%, and 0.11% in all-cause, cardiovascular, and respiratory premature deaths, respectively^20,21^. Similarly, for MDA8 O_3_, each 1 μg m^−3^ rise in its concentration was associated with increases of 0.10% in non-accidental mortality, 0.15% in cardiovascular mortality, and 0.20% in respiratory mortality^22^. It's important to note that the β values used in this study to assess human health risks due to PM_2.5_ and O_3_ are derived from the above these studies. Referring to the study from Zhao et al.^16^, PM_2.5_ and MDA8 O_3_ indicators were employed to estimate premature deaths across three health endpoints attributed to long-term exposure to PM_2.5_ and O_3_. The calculation formulas are as follows:

1 $${\text{RR}} = \exp \, \left[ {\beta \times \left( {{\text{C}} - {\text{C}}_{0} } \right)} \right]$$

2 $${\text{E}} = \left[ {\left( {{\text{RR}} - {1}} \right)/{\text{RR}}} \right] \times {\text{P}} \times {\text{F}}_{{\text{p}}} = \left[ {{1} - {\text{exp}}^{{ - \beta \times ({\text{C }} - {\text{ C}}0)}} } \right] \times {\text{P}} \times {\text{F}}_{{\text{p}}}$$

Here, C represents the annual average concentration of PM_2.5_ and MDA8 O_3_, while C_0_ denotes the safety threshold. If the concentration exceeds C_0_, it signifies potential health risks. The C_0_ values for PM_2.5_ and MDA8 O_3_ are set at 10 μg m^−3^ and 26.7 ppb, respectively, based on the study by Kuerban et al.^23^. β represents the percentage increase in health effects associated with a 1 μg m^−3^ increase in PM_2.5_ and MDA8 O_3_ concentration. As previously mentioned, β values for all-cause and cardiovascular mortality attributed to PM_2.5_ are 0.34% (95% CI 0.08–0.50%) and 0.07% (95% CI 0.04–0.09%), respectively^20,21^. For MDA8 O_3_, corresponding β values are 0.10% (95% CI 0.05–0.20%) and 0.15% (95% CI 0.05–0.29%), respectively^22^. The β values of respiratory mortality are 0.11% (95% CI 0.00–0.22%) for PM_2.5_ and 0.20% (95% CI 0.00%, 0.43%) for MDA8 O_3_^22,24^. RR represents the relative risk, while P denotes the exposed population of each city. F_p_ denotes the mortality rate for three health endpoints. According to the study by Liao et al.^25^ on municipal-level mortality rates, where detailed information regarding the F_p_ of each city from 2006 to 2012 was provided. Considering the minimal fluctuation in F_p_ values each year, we utilized the average F_p_ value from their study covering the years 2006 to 2016 as the F_p_ value for calculating the health impacts of PM_2.5_ and O_3_ during 2015–2019 in this study, as shown in Table 1. E represents the number of deaths related to PM_2.5_ and O_3_.

**Table 1:** The F_p_ value for all-cause, cardiovascular, and respiratory in each city of GBA.

Region	F_p_ for all-cause (‰)	F_p_ for cardiovascular (‰)	F_p_ for respiratory (‰)
Guangzhou	5.35	1.74	1.26
Shenzhen	1.33	0.51	0.32
Zhuhai	3.20	1.09	0.50
Foshan	5.34	1.55	1.03
Jiangmen	7.49	2.28	1.28
Zhaoqing	6.81	1.72	1.84
Huizhou	6.18	1.94	1.10
Zhongshan	5.89	1.75	0.86
Dongguan	4.77	1.47	0.84
Hong Kong	5.53	1.35	1.05

## Results and discussion

### Spatiotemporal distribution and monthly variation of PM_2.5_ and MDA8 O_3_

**Figure 2:**
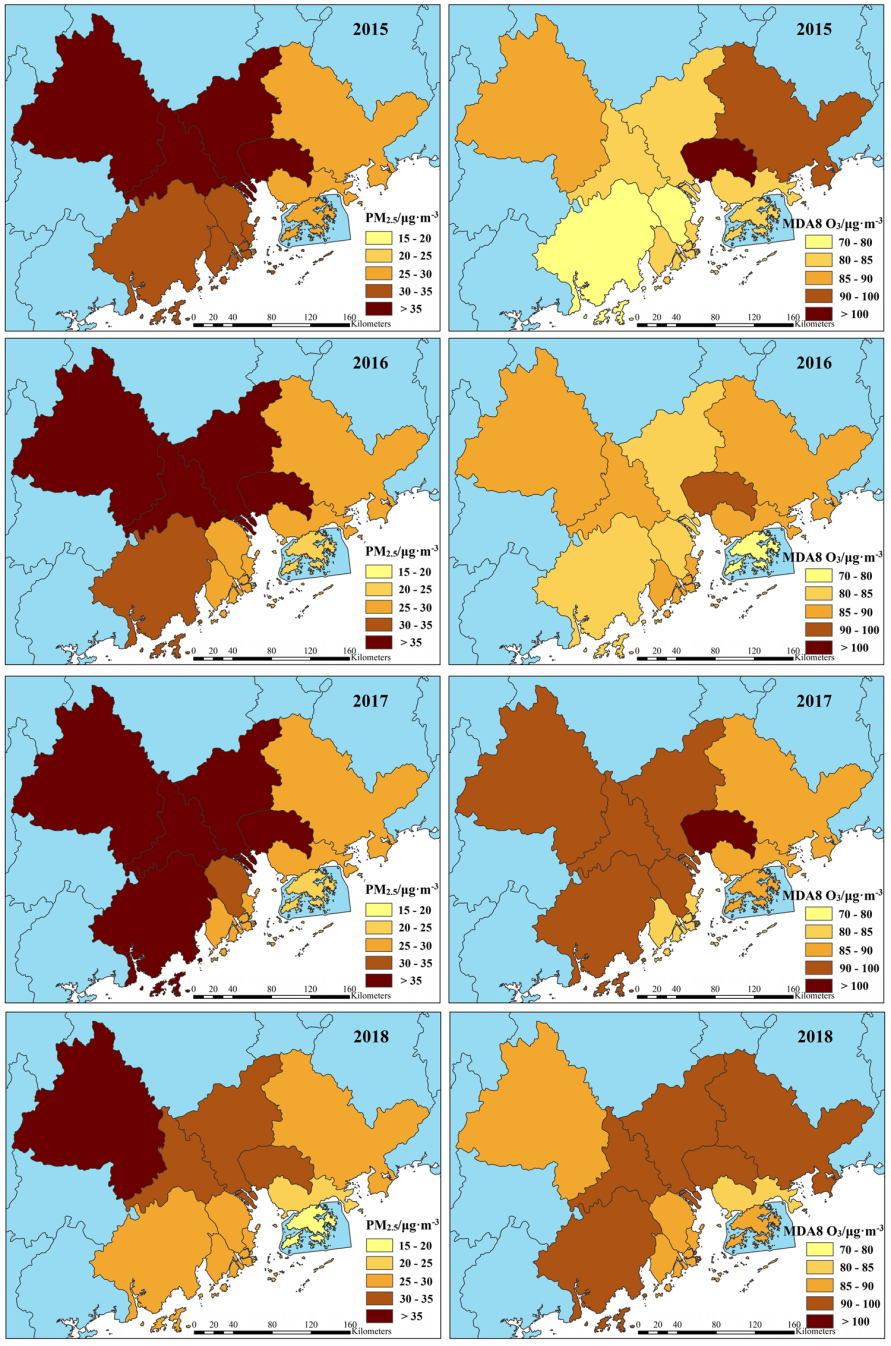

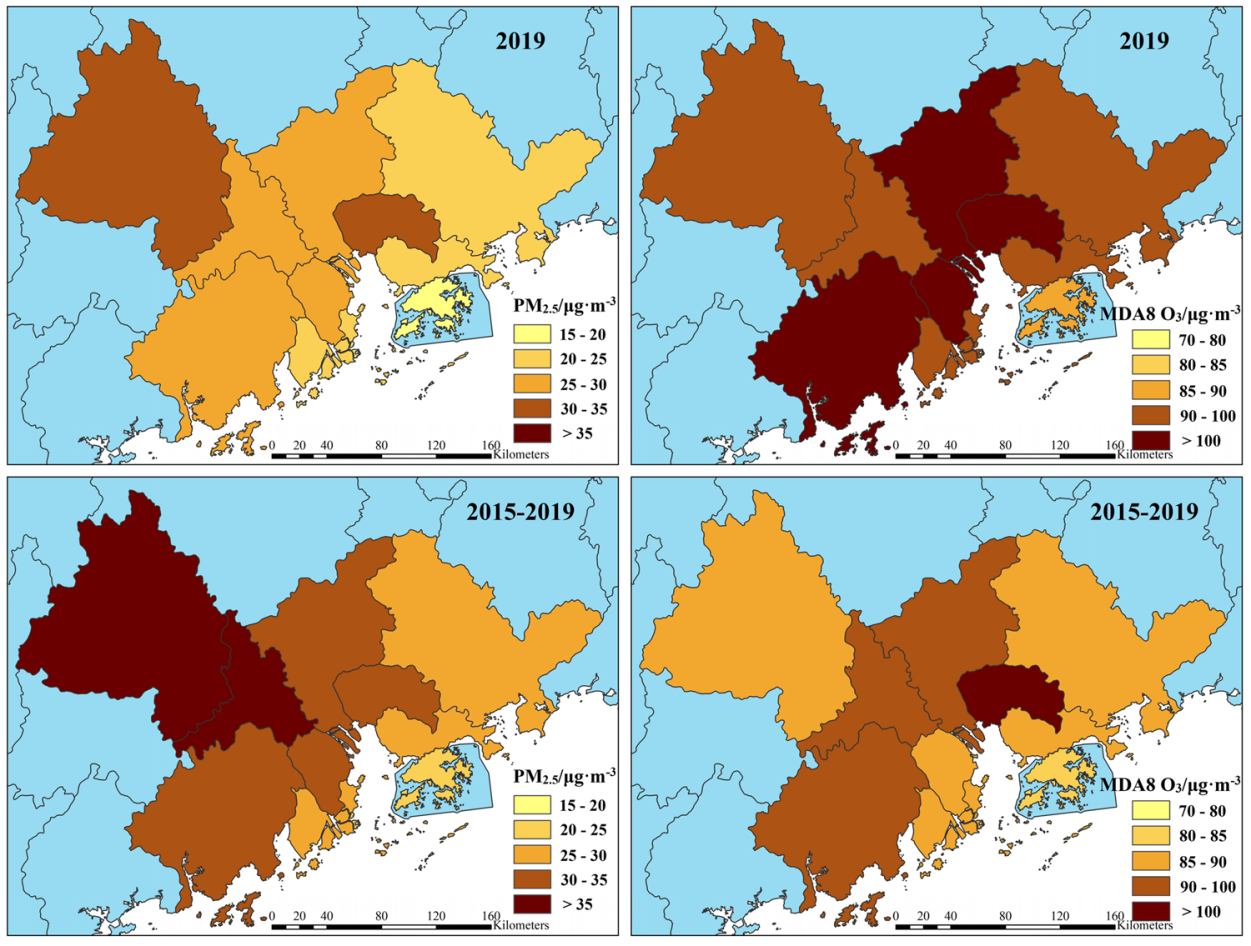
Spatio-temporal distribution of PM_2.5_ and MDA8 O_3_ in each city from 2015 to 2019 (The map was generated by ArcGIS 10.7 https://www.esri.com/en-us/arcgis/products/arcgis-desktop/resources).

Figure 2 indicates the spatial pattern and average concentrations of PM_2.5_ and MDA8 O_3_ across various cities in the GBA over the five-year period. Overall, there was a gradual decrease in PM_2.5_ concentration in each city from 2015 to 2019. Additionally, the spatial distribution of PM_2.5_ remained consistent each year, exhibiting a pattern of decrease from northwest to southeast, which was consistent with previous findings by Lin et al.^26^ and Miao et al.^27^ utilizing satellite remote sensing technology. The highest PM_2.5_ concentration was recorded in Zhaoqing (31.8–40.4 μg m^−3^), followed by Foshan (29.8–39.6 μg m^−3^) and Dongguan (31.9–37.1 μg m^−3^). This phenomenon could be attributed to the fact that these cities are inland and the presence of mountains obstructs the dispersion of PM_2.5_^9^. The concentrations in square brackets represent the maximum and minimum values of PM_2.5_ during 2015–2019. However, coastal cities benefit from ocean breezes, which facilitate the dispersion and dilution of PM_2.5_. Furthermore, higher levels of precipitation aids in the deposition of PM_2.5_, thereby reducing its concentration in the air. Therefore, the concentration of PM_2.5_ in coastal cities such as Hong Kong (18.9–27.0 μg m^−3^), Macao (17.4–29.3 μg m^−3^), Shenzhen (24.1–29.8 μg m^−3^), and Huizhou (24.8–29.5 μg m^−3^) was relatively low. A similar phenomenon was also observed in the study by Fang et al.^18^.

MDA8 O_3_ presented the opposite spatiotemporal distribution compared to PM_2.5_, with its concentration generally increasing in each city during the period between 2015 and 2019. Spatially, MDA8 O_3_ concentration exhibited a decreasing pattern from east to west. Higher concentrations were observed in Dongguan (92.3–110.7 μg m^−3^), foshan (81.5–99.8 μg m^−3^), and Jiangmen (73.2–104.2 μg m^−3^), whereas some cities like Hong Kong (77.5–88.8 μg m^−3^) and Shenzhen (80.3–93.8 μg m^−3^) had lower concentrations. This disparity may be attributed to higher temperatures, stronger photochemical reactions, and larger emissions of O_3_ precursors such as NO_x_ and VOC_s_ from ships and ports in coastal cities^10^.

**Figure 3:**
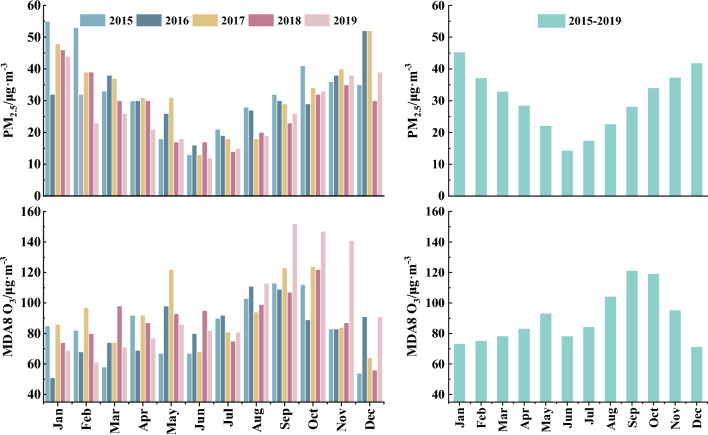
Monthly variation characteristics of PM_2.5_ and MDA8 O_3_ in the GBA.

To investigate the seasonal variations of PM_2.5_ and O_3_, Fig. 3 illustrates their monthly average concentrations over the five-year period. While PM_2.5_ exhibits minor fluctuations across different months and years, its monthly pattern generally resembles a “V” shape, with higher concentrations in winter (Dec., Jan. and Feb.) and autumn (Sep., Oct. and Nov.), and lower concentrations in summer (Jun., Jul. and Aug.) and spring (Mar., Apr. and May.). The highest concentration of PM_2.5_ occurs during winter, which was attributed to increased anthropogenic emissions and unfavorable meteorological conditions^17^. Lower temperatures, reduced light intensity, shorter sunshine duration, and stable atmospheric stratification in winter facilitate the formation of a strong and persistent inversion layer. This inhibits the diffusion and dilution of PM_2.5_, leading to its continuous accumulation in the air and frequent heavy pollution events^9^. PM_2.5_ levels begin to decline from January, reaching their lowest point in June, and gradually increase thereafter until December. During summer, PM_2.5_ concentrations remain low due to factors such as intense solar radiation, strong atmospheric convection, and a thinner temperature inversion layer, which collectively enhance air ventilation and PM_2.5_ dilution. Additionally, summer weather is typically rainy, and the wet deposition of particulate matter, along with cleaner air brought by marine monsoon, contributes to the removal of PM_2.5_^11^.

Studies have suggested that the concentration of O_3_ in southern cities was significantly higher than that in northern cities in China^9^. In northern cities, the monthly variation of MDA8 O_3_ formed an inverted V shape, with the highest concentration occurring around June^8^. Conversely, in southern cities, it exhibited a distinctive M-shaped pattern, peaking in May–June and then gradually decreasing, with a second peak in September-October^8,9^, which is consistent with the findings of this study. Regarding seasons, higher MDA8 O_3_ levels were observed in autumn, followed by summer, spring, and winter. Surface O_3_ is primarily produced through the photochemical reaction of precursors, a process whose rate is influenced by various meteorological conditions, including temperature, solar radiation, relative humidity, and precipitation^10^. Typically, during summer, characterized by high temperatures, ample sunshine, and dry air, the photochemical reaction of O_3_ precursors intensifies, facilitating the formation of O_3_. However, our study reveals a noteworthy finding: the peak MDA8 O_3_ concentration occurred in September during autumn, rather than in summer. This was attributed to the frequent precipitation in summer, which effectively inhibited the production of O_3_^2^. The lowest MDA8 O_3_ concentrations were observed in winter. Firstly, colder temperatures and weaker sunlight reduce the occurrence of photochemical reactions that generate O_3_. Additionally, atmospheric stability in winter hinders the mixing and dispersion of O_3_. Moreover, emissions of O_3_ precursors like VOC_s_ from plants may decrease in winter, further limiting O_3_ formation. Overall, these factors contribute to lower O_3_ concentrations during the winter months^10^.

### Change trends of PM_2.5_ and MDA8 O_3_ during 2015–2019

**Figure 4:**
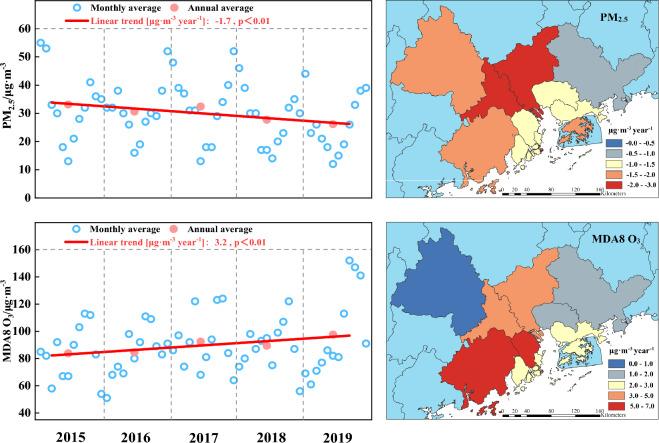
Linear change trend of PM_2.5_ and MDA8 O_3_ in five years (The map on the right was generated by ArcGIS 10.7 https://www.esri.com/en-us/arcgis/products/arcgis-desktop/resources).

Figure 4 illustrates the trend analysis of the two pollutants in the GBA and its corresponding cities. The annual average PM_2.5_ concentrations in this area from 2015 to 2019 were 33.1 μg m^−3^, 30.6 μg m^−3^, 32.4 μg m^−3^, 27.7 μg m^−3^, and 26.1 μg m^−3^, respectively, indicating an overall downward trend. Linear fitting based on the average concentration of each year over the five-year period revealed a decline rate for PM_2.5_ in this region of 1.7 μg m^−3^/year. Among the eleven cities analyzed, Macao (-2.8 μg m^−3^/year), Foshan (-2.5 μg m^−3^/year), Guangzhou (-2.1 μg m^−3^/year), Jiangmen (-2.0 μg m^−3^/year), and Hong Kong (-1.9 μg m^−3^/year) exhibited higher decline rates over the five-year period. Conversely, Huizhou (-0.6 μg m^−3^/year) and Dongguan (-1.0 μg m^−3^/year) experienced lower declines. By 2019, although the annual average PM_2.5_ concentration of all cities in the GBA fell below the level-2 Chinese Ambient Air Quality Standard (CAAQS, GB3095-2012) threshold of 35 μg m^−3^, none of the cities had yet achieved the Grade I annual standards (15 μg m^−3^) specified in the CAAQS. Thus, PM_2.5_ pollution remains a significant concern in this region, necessitating the implementation of more stringent air pollution control measures to enhance air quality.

Contrary to PM_2.5_, MDA8 O_3_ in the GBA has generally exhibited an upward trend over the past five years, with concentration of 83.8 μg m^−3^ in 2015, 84.6 μg m^−3^ in 2016, 92.4 μg m^−3^ in 2017, 89.4 μg m^−3^ in 2018, and 97.6 μg m^−3^ in 2019. The average rise of MDA8 O_3_ over the period 2015–2019 was 3.2 μg m^−3^/year. Notably, the upward trend was particularly evident in eight cities, except for Zhaoqing (+ 1.0 μg m^−3^/year), Huizhou (+ 1.3 μg m^−3^/year), and Dongguan (+ 1.8 μg m^−3^/year). Specifically, significant increases were observed in Jiangmen (+ 6.9 μg m^−3^/year), Zhongshan (+ 5.8 μg m^−3^/year), Guangzhou (+ 4.5 μg m^−3^/year), and Foshan (+ 4.0 μg m^−3^/year). Consistent with our findings, previous studies have also indicated a shift in China's main air pollutant from PM_2.5_ to O_3_ since 2013^19,28^. Indeed, there exists a correlation between the overall decline in PM_2.5_ and the rise in O_3_. Li et al.^19^ demonstrated that a key factor contributing to the increase in summer O_3_ in the North China Plain during 2013–2017 was the decrease in PM_2.5_, which enhanced surface solar radiation and facilitated atmospheric photochemical reactions, thereby exacerbating O_3_ pollution. However, these relationships are not purely causal, as the increase in O_3_ is complex and influenced by various factors such as meteorological conditions, emission sources, and chemical reactions^17^. Consequently, while future efforts should prioritize controlling PM_2.5_, the government should also closely control O_3_ pollution in this region.

Numerous studies have documented changes in PM_2.5_ and O_3_ pollution across various regions of China in recent years. In Beijing, for the period 2014–2018, PM_2.5_ levels were observed to decrease while MDA8 O_3_ levels were on the rise, with rates of change measured at 7.4 μg m^−3^/year and 1.3 μg m^−3^/year, respectively^29^. Similarly, in the YRD region during the same period, Zhao et al.^17^ reported a decline in PM_2.5_ and an increase in MDA8 O_3_ by 3.1 μg m^−3^/year and 3.6 μg m^−3^/year, respectively. In the BTH region, the rates of change were even more pronounced at 7.1 μg m^−3^/year for PM_2.5_ decrease and 5.4 μg m^−3^/year for MDA8 O_3_ increase. Contrastingly, in the PRD region, PM_2.5_ exhibited a decreasing trend of 2.2 μg m^−3^/year from 2015 to 2020, while MDA8 O_3_ increased by 1.8 μg m^−3^/year^30^. While the decline rate of PM_2.5_ in the other 9 cities except Macao and Foshan was much lower than that observed in Beijing, YRD, and BTH regions, and the rising rate of MDA8 O_3_ was comparatively lower than in the YRD and BTH regions, it is noteworthy that MDA8 O_3_ levels in the GBA still reached nearly 100 μg m^−3^ in 2019, significantly exceeding the national average level. These findings underscore the imperative for further improvements in PM_2.5_ and O_3_ levels in the GBA.

### Health impact assessment of PM_2.5_ and O_3_

Since the establishment of air quality monitoring stations in 2013, previous studies have extensively assessed the health impacts of air pollution across China^15–17,22–24^. A study conducted by Kuerban et al.^23^ found the numbers of premature deaths, cardiovascular diseases, respiratory diseases, and chronic bronchitis attributed to long-time PM_2.5_ exposure in China for the year 2018 were 334,118, 70,983, 109,327, and 228,855, respectively. They decreased by 23%, 25%, 27%, and 27%, respectively, compared to 2015, reflecting China's achievements in controlling health risks from PM_2.5_ in recent years. Regarding long-term exposure to O_3_ in 2019, predictions indicated that health impacts estimates on all-cause mortality, respiratory mortality, and cardiovascular mortality were 181,000 (95% CI 91,500–352,000), 33,800 (95% CI 0–71,400), and 112,000 (95% CI 38,100–214,000), respectively, which increased by 53%, 55%, and 53%, respectively, compared to the year 2015^22^. While these studies have significantly contributed to our understanding of PM_2.5_ and O_3_ health risk assessment in China, it's essential to acknowledge that they uniformly applied the same mortality rate (F_p_) for health endpoints across all cities in China, potentially reducing the reliability of the evaluation results. Given the differences in F_p_ values for health endpoints across different cities, the utilization of municipal-level F_p_ values for health endpoints in this study could yield a more accurate health risk estimate compared to previous studies.

**Figure 5:**
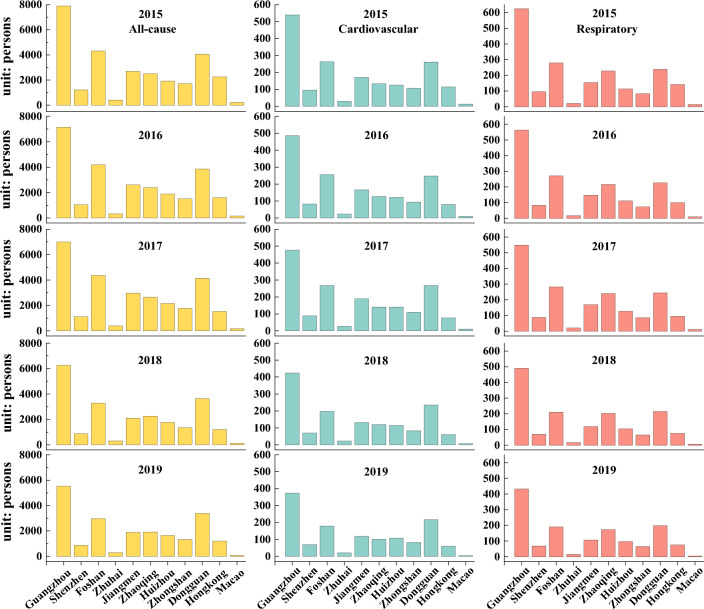
The estimated PM_2.5_-related health impacts in various cities during 2015–2019.

Table 2 shows that the total PM_2.5_-related all-cause mortality, cardiovascular diseases, and respiratory diseases in the GBA in 2019 were 21,113 (95% CI 4968–31,048), 1333 (95% CI 762–1714), and 1424 (95% CI 0–2848), respectively, indicating decreases of 27.6%, 28.0%, and 28.4%, respectively, compared to 2015. At the municipal level, the highest percentage decrease in PM_2.5_-attributed all-cause mortality from 2015 to 2019 was observed in Macao (60.9%), followed by Hong Kong (46.6%) and Foshan (31.2%), suggesting that the control of PM_2.5_ has brought better health benefits for them._._ Health effects associated with PM_2.5_ mainly depend on PM_2.5_ concentrations and population size. As a result, certain cities with high levels of PM_2.5_ and population density, such as Foshan [all-cause deaths (AD): 2963–4367; cardiovascular deaths (CD): 179–267; respiratory deaths (RD): 190–282], and Dongguan [AD: 3385–4147; CD: 217–268; RD: 198–244], have exhibited a significant number of deaths (see Fig. 5). Note that the range in brackets indicates the number of deaths from 2015 to 2019. Although Zhaoqing [AD: 1921–2648; CD: 101–141; RD: 173–240] had the highest concentration of PM_2.5_ among all cities, its population is relatively small compared to others, resulting in a lower number of deaths caused by PM_2.5_. Similarly, while Guangzhou's [AD: 5523–7901; CD: 373–540; RD: 432–624] PM_2.5_ concentration is not exceptionally high, its population exceeds 16 million, making it the city with the greatest health risk from PM_2.5_ exposure. Furthermore, despite Shenzhen's [AD: 869–1208; CD: 69–96; RD: 69–96] larger population, its PM_2.5_ pollution levels are relatively lower, resulting in reduced health risks related to PM_2.5_ exposure.

**Table 2:** Estimation of the impact of PM_2.5_ and O_3_ on human health in the GBA.

Air pollutant	Mortality	2015	2016	2017	2018	2019
PM_2.5_	All-cause (min–max)	29,169 (6863–42,895)	26,696 (6281–39,258 )	28,224 (6641–41,505)	23,130 (5442–34,015)	21,113 (4968–31,048)
	Cardiovascular (min–max)	1852 (1058–2382)	1694 (968–2178)	1793 (1025–2306)	1464 (836–1882)	1333 (762–1714)
	Respiratory (min–max)	1989 (0–3979 )	1817 (0–3634)	1913 (0–3826)	1572 (0–3144)	1424 (0–2848)
O_3_	All-cause (min–max)	11,161 (5581–22,323)	11,261 (5631–22,522)	14,400 (7200–28,801)	13,034 (6517–26,068)	16,286 (8143–32,572)
	Cardiovascular (min–max)	5009 (1670–9685)	5070 (1690–9803)	6456 (2152–12,482)	5851 (1950–11,312)	7321 (2440–14,155)
	Respiratory (min–max)	4379 (0–9414)	4409 (0–9479)	5592 (0–12,023)	5078 (0–10,918)	6314 (0–13,576)

**Figure 6:**
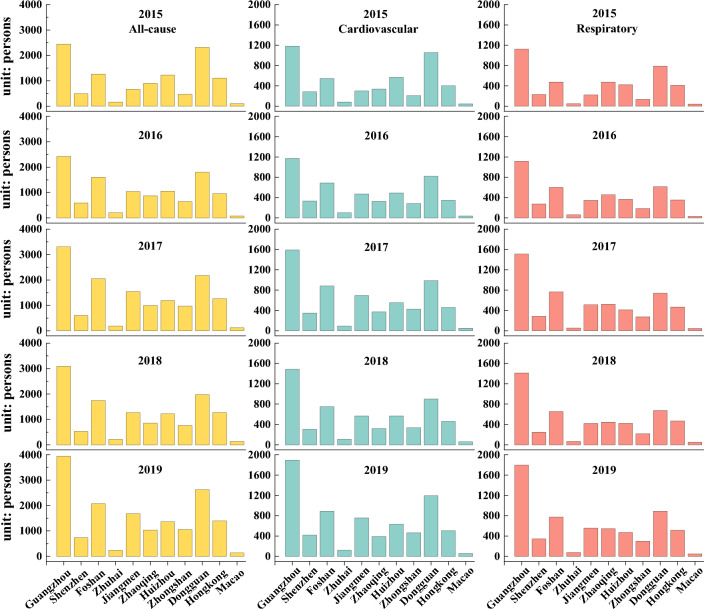
The estimated O_3_-related health impacts in various cities during 2015–2019.

On the contrary, the all-cause, cardiovascular and respiratory-related deaths due to long-time O_3_ exposure increased by 45.9% from 11,161 (95% CI 5581–22,323) to 16,286 (95% CI 8143–32,572), 46.2% from 5009 (95% CI 1670–9685) to 7321 (95% CI 2440–14,155), and 44.2% from 4379 (95% CI 0–9414) to 6314 (95% CI 0–13,576), respectively, during 2015–2019 (Table 2). Spatially, a significant percentage increase (> 30%) in all-cause deaths were observed in Guangzhou (61.3%), Jiangmen (41.6%), and Foshan (33.0%), highlighting the urgent need for implementing O_3_ control measures in these cities. However, the increases in the mortality burden of diseases attributable to O_3_ were not significant in Macao (1.0%) and Zhuhai (3.1%). Similar to PM_2.5_, Guangzhou [AD: 2423–3941; CD: 1170–1894; RD: 1114–1796], Dongguan [AD: 1799–2628; CD: 821–1194; RD: 616–891], Foshan [AD: 1266–2074; CD: 546–890; RD: 477–774], etc. had a higher number of O_3_-related deaths due to their large population, as shown in Fig. 6. Although Shenzhen had a larger population, its O_3_ concentration was lower, so it had only 495–738 AD, 282–419 CD, and 233–345 RD, because its F_p_ values and O_3_ concentration were very low. In addition, Jiangmen [AD: 665–1682; CD: 301–756; RD: 223–555] had relatively high O_3_ levels despite its small population, resulting in a comparatively high O_3_ risk. Conversely, Hong Kong[AD: 956–1394; CD: 347–504; RD: 355–515], with a larger population but the lowest O_3_ concentration among all cities, also experienced higher risks associated with O_3_.

Exposure–response coefficient (β) and safety threshold (C_0_) are critical factors in air pollution risk assessment. In China, due to the absence of comprehensive cohort studies on long-term exposure to PM_2.5_ and O_3_, there is no uniform determination of β values^20^. Consequently, different epidemiological studies have employed varying β values, leading to differing estimations of health risks^31–33^. For example, Feng et al.^34^ and Zhang et al.^12^ used different β values to estimate total mortality attributed to PM_2.5_ in China for 2015, reporting 1,130,000 and 1,850,000 deaths, respectively. Additionally, Zhang et al.^24^ utilized β values from Zhang et al.^21^ and Yin et al.^14^ to calculate 205,800 (95% CI 176,200–240,000) and 121,500 (95% CI 66,200–176,221) AD caused by O_3_ exposure across China in 2015, respectively. Currently, there is no theoretical explanation for C_0_. Regarding C_0_ for PM_2.5_, the World Health Organization (WHO) has recommended a reference concentration of 10 μg m^−3^^14,35^. Regarding the O_3_ threshold value for short-term exposure, currently, there is no theoretical explanation. The WHO has recommended 35 ppb (70 μg m^−3^) as the baseline level of O_3_^22^, while a threshold of 100 μg m^−3^ has been deemed safe for public health by both the CAAQS Grade I and WHO^23^. In previous studies on the health risks of long-term O_3_ exposure, epidemiological studies have confirmed that a threshold value of 26.7 ppb has the highest correlation with disease mortality^22^. Therefore, this study utilized it to evaluate the health risks of long-term O_3_ exposure. When employing the same β and C_0_ values as used in our study, Kuerban et al.^23^ estimated that the total AD attributed to PM_2.5_ in the 9 cities of the PRD were 20,306 and 18,877 in 2015 and 2018, respectively, which were lower than our evaluation results. However, their estimates for both CD and RD were notably higher than ours. A similar discrepancy was observed when comparing the estimation of human health risks caused by O_3_ in our study with the results of Zhao et al.^16^. One potential explanation for this disparity between these studies is that our study utilized city-level F_P_ values, whereas their studies employed the national average F_P_ value for each city^16^.

It is imperative to acknowledge that this study entails certain uncertainties. On the one hand, the acquisition of mortality data for various cities presents challenges, leading us to rely on the average F_p_ values reported by Liao et al.^25^ for the years 2006–2012 to assess the health impacts of PM_2.5_ and O_3_ in GBA from 2015 to 2019. Despite the minimal annual fluctuations in F_p_ values per city, they could still impact the estimation results of this study. On the other hand, the current distribution of air quality monitoring stations predominantly focuses on urban areas within the GBA and is limited in number. In this study, the annual average concentrations of PM_2.5_ and O_3_ for each city were determined by averaging the data from all monitoring stations within that city, which could also affect the accuracy of the assessment results to some extent. While spatial interpolation techniques offer insights into the spatial distribution of pollutant concentrations, their efficacy is constrained by the scarcity of monitoring stations. Additionally, the absence of crucial data such as the number of disease-related deaths across different hospitals and the spatial distribution of cases significantly impacts the estimation of health mortality. In summary, to more accurately assess the impact of atmospheric pollution on human health, it is necessary for future research to establish more air quality monitoring stations in the region. Additionally, the utilization of more precise disease data can help mitigate this uncertainty.

## Conclusions

This is the first study to comprehensively assess combined pollution characterized by PM_2.5_ and O_3_ and its potential health impacts in the GBA. We observed a decline in PM_2.5_ and a rise in MDA8 O_3_ during 2015–2019, with a decline rate for PM_2.5_ of 1.7 μg m^−3^/year and a rise rate for MDA8 O_3_ of 3.2 μg m^−3^/year. The significant decrease in PM_2.5_, particularly in Macao, Foshan, Guangzhou, and Jiangmen, highlights the efforts of these cities in controlling PM_2.5_ in recent years. On the other hand, Jiangmen exhibited the highest increase in MDA8 O_3_, followed by Zhongshan and Guangzhou, indicating the urgent need to implement measures to prevent O_3_ pollution in these regions in the future. Compared to 2015, the estimated number of AD, CD, and RD in 2019 caused by PM_2.5_ decreased by 27.6%, 28.0%, and 28.4%, respectively. In contrast, those caused by O_3_ increased by 45.9%, 46.2%, and 44.2%, respectively. These findings indicate that the health benefits resulting from improvements in PM_2.5_ might be offset by the health risks associated with increased O_3_ levels if insufficient attention is given to O_3_ control in the future. Thus, it is urgent to implement coordinated control of PM_2.5_ and O_3_ in the GBA.

## Data Availability

Te datasets are not publicly available due to data privacy but are available from the corresponding author on reasonable request.
